# Cord Blood Leptin Levels of Healthy Neonates Are Associated with IFN-γ Production by Cord Blood T-Cells

**DOI:** 10.1371/journal.pone.0040830

**Published:** 2012-07-16

**Authors:** Athanasia Mouzaki, Ioannis Panagoulias, George Raptis, Evagellia Farri-Kostopoulou

**Affiliations:** 1 Division of Hematology, Department of Internal Medicine, Medical School, University of Patras, Patras, Greece; 2 Royal Oldham Hospital, Department of Child Health, Oldham, United Kingdom; 3 Neonatal Intensive Care Unit, St Andrew’s General Hospital of Patras, Patras, Greece; University of Cambridge, United Kingdom

## Abstract

Leptin is a hormone synthesized by adipocytes and other tissues, including the placenta, and it regulates food intake and energy expenditure, reproductive and immune functions. To investigate the role of leptin in neonatal immunity, we measured serum leptin and cytokine (IFN-γ, TNF-α, IL-2, IL-4, IL-10, IL-12) levels in the cord blood (cb) of 510 healthy neonates, 14 small for gestational age (SGA), 312 appropriately grown for gestational age (AGA) and 184 large for gestational age (LGA). Median serum leptin concentration in the whole sample was 11 ng/ml. In 11.2% neonates (1 SGA, 32 AGA, 24 LGA), leptin levels were >90th percentile (median 39 ng/ml). In 33.3% of those (3.72% of total sample) with the highest leptin levels (median 46 ng/ml), significantly elevated levels of serum IFN-γ were also found (mean 27.11 pg/ml, range 17.5–38.5 pg/ml). In neonates with leptin levels ∼50th percentile (median 12 ng/ml) or <10th percentile (median 1 ng/ml), serum IFN-γ levels were negligible. All other cytokines measured, were < the assays’ detection limits. To investigate whether leptin can independently influence cytokine gene expression by cb T-cells and monocytes (Mc), we cultured cb T-cells or Mc, isolated from randomly selected AGA neonates or adult peripheral blood, with leptin. This resulted in upregulation of IL-2, IFN-γ and IL-4 gene expression in cb and adult T-cells and IL-10 expression mainly in cb-Mc. Significantly higher expression of IFN-γ occurred in female cb-T-cells cultured with leptin, compared with male cb-T-cells. In conclusion, the concurrent presence of high concentrations in both leptin and IFN-γ in cb of healthy infants, and leptin’s ability to directly upregulate cytokine gene expression in cb T and Mc cells, indicate that abnormally high leptin levels can independently influence the immune system of healthy newborns, and may mediate gender differences in the development of a Th1 polarized immune response.

## Introduction

Leptin, a 16 kDa non-glycosylated polypeptide product of the obese (ob) gene [1], is an adipocyte-derived hormone that participates in the regulation of energy homeostasis [2], neuroendocrine function [3], angiogenesis [4], [5], bone formation [6] and reproduction [7]. The central action of leptin, is mediated by binding to its hypothalamic receptors [8], [9]. Leptin is structurally similar to long-chain helical cytokines [10], [11] and it signals through its receptor, OB-R [11], which is a member of the cytokine receptor superfamily. Leptin is mainly expressed in the adipose tissue, but also in the placenta, bone marrow, stomach, brain, muscle and fetal tissues [12], [13]. In pregnancy, leptin regulates fetal growth, placental angiogenesis and mobilization of maternal fat [13]. Serum leptin levels in pregnant women are significantly higher than in non-pregnant women [14]. The human placenta expresses high amounts of leptin mRNA and protein in early, mid, and late gestation [14], [15]. Adipocytes have not been found in placental tissue [16].

There is no correlation between maternal leptin levels and fetal weight, whereas several studies have reported that umbilical cord blood leptin levels correlate with fetal insulin, birth weight, ponderal index, length and head circumference [17], [18], [19]. The higher leptin levels in umbilical veins compared with umbilical arteries, and the marked fall during the neonatal period, indicate that the human placenta is a major source of leptin in the fetal circulation [20].

There is growing evidence that leptin regulates the maturation of the fetal and neonatal immune system: Mice lacking leptin or its functional receptor have a number of defects in both cell-mediated and humoral immunity [21], [22]. Similarly, humans with congenital leptin deficiency have a much higher incidence of infection-related death during childhood [23]. In the latter, administration of exogenous leptin reverses their abnormal immunophenotype, T-cell hyporesponsiveness and Th2 cytokine production [24].

Leptin affects both the endocrine and immune systems, and there is functional connection and anatomical contiguity between adipocytes and lymphoid cells [25]. In innate immunity, leptin seems to promote the activation of monocytes, dendritic cells and macrophages, and stimulates them to produce pro-inflammatory cytokines [26], [27] or the anti-inflammatory cytokine IL-10 [28], [29]. In NK cells, leptin is involved in all processes of cell development, differentiation, proliferation, activation, and cytotoxicity [30]. Leptin has been also shown to modulate the adaptive immunity by enhancing T-cell survival, promoting the proliferation of naive but not memory T-cells, and stimulating the production of the Th0/Th1 cytokines IL-2 and IFN-γ [31].

The pro-Th1 immunomodulatory effects of leptin have been linked to enhanced susceptibility to autoimmune or inflammatory diseases, mainly in experimental models [32], [33], [34], [35]. In addition, leptin was shown to inhibit the proliferation of human PBMC-derived natural T regulatory cells [36]. In human autoimmune patients, elevated serum leptin levels occur in certain diseases and may play a causal role in the disease progress [37], [38], [39] although a direct link of leptin to human autoimmune disease pathogenesis has not been yet established [28], [40].

To investigate the role of leptin in neonatal immunity, we measured leptin levels in cord blood of healthy newborns and tested for a possible correlation with cytokine levels. In addition, we studied whether leptin has a direct effect on the expression of pro- and anti-inflammatory cytokine genes in cord blood mononuclear cells, and purified cord blood T-cells and monocytes.

We observed that in a small percentage of neonates leptin levels were very high, and in those neonates IFN-γ levels were also significantly elevated. Culture with leptin, upregulated cytokine gene expression in T-cells and monocytes. In addition, we observed that higher expression of IFN-γ occurred in T-cells derived from the cord blood of female neonates.

## Results

### Data of Study Subjects

The demographic characteristics of the 510 neonates are shown in Table 1. The anthropometric characteristics of the mothers are shown in Table 2 and of the neonates in Table 3. Group statistics showed that in each group of infants (SGA, AGA, LGA) male and female neonates had similar somatometric characteristics and PI at birth.

**Table 1:** Demographic characteristics of the neonates*.

Variables		n	%
Sex	Males	253	49.6
	Females	275	50.3
Race	White	487	95.5
	Asian	23	4.5
Birth weight & gestational age	SGA	14	2.7
	AGA	312	61.2
	LGA	184	36.1

* Data are presented as the frequency count and percentages.

**Table 2:** Anthropometric characteristics of the mothers*.

Variables	Mean (SD)	Min.	Max.
Age (yrs)	27.19 (4.97)	15	42
Height (cm)	164.03 (6.41)	146	180
Weight before pregnancy (kg)	62.93 (12.59)	40	124
Weight at birth (kg)	77.57 (12.88)	50	137

* Data are presented as mean (± standard deviation) with minimum and maximum values.

**Table 3:** Anthropometric characteristics of the newborns#.

		Males			Females		
Variables		SGA n = 2	AGA n = 149	LGA n = 102	SGA n = 12	AGA n = 163	LGA n = 82
Birth weight (g)	Min	2380	2420	3530	2160	2350	3550
	Max	2390	3780	4650	2580	3750	5200
	Mean	2385.0	3242.7	4015	2401.7	3113.2	4097.9
	SD	7.1	267.1	273.4	163.9	303.8	380.6
Length (cm)	Min	45	46	48	44	45	49
	Max	46	55	58	48	55	59
	Mean	45.5	50.8	53.1	46.7	50.4	53.2
	SD	0.7	1.9	2.0	1.2	1.8	2.0
PI	Min	3	2	2	2	2	2
	Max	3	3	4	3	3	3
	Mean	3	2.4	2.7	2.2	2.4	2.8
	SD	0.0	0.5	0.5	0.4	0.5	0.4
Head circ. (cm)	Min	31	32	32	33	31	34
	Max	33	38	38	34	37	38
	Mean	32	34.7	35.9	33.2	34.0	35.9
	SD	1.4	1.1	1.3	0.4	1.1	1.0

# Summary statistics calculated separately for the subgroups SGA, AGA and LGA. Data are presented as mean (± standard deviation) with minimum and maximum values. PI, ponderal index: birthweight (g) ×100**/**height (cm)^3^; Head circ., head circumference.

### Leptin Levels of the Neonates

**Figure 1:**
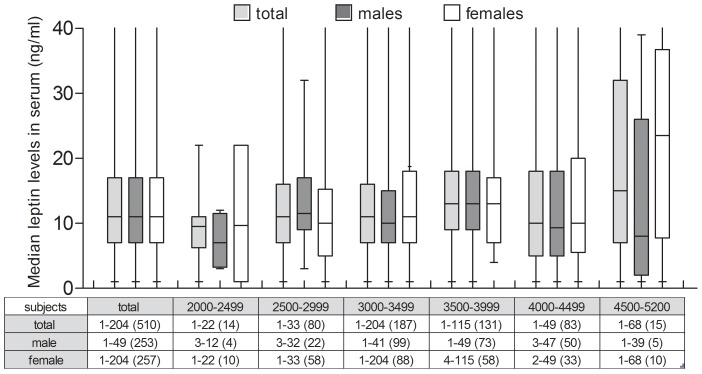
Cord blood leptin levels in healthy neonates categorized by birth weight. Median serum leptin levels in 7 weight categories (g); each includes leptin values for the total sample, male and female infants. The table shows the min. and max. leptin values (ng/ml) and the number of subjects (n) per weight group (total, males, females).

Serum leptin levels of the neonates are shown in Table 4 and a detailed analysis of median leptin levels vs birth weight of the neonates (presented as total, males and females/weight group) is shown in Figure 1. Leptin was detected in all samples. The median cord blood leptin concentration was 11 ng/ml (range 1–204 ng/ml). There was no significant correlation between the anthropometric characteristics of the mothers (Table 2) and cord blood leptin levels of the newborns (multiple linear regression analyses among 510 term or near-term pregnancies).

**Table 4:** Leptin levels of the total neonatal population*.

Median (ng/ml)	11
Minimum	1
Maximum	204
Percentiles (%)	
10	4.8
20	7
50	11
90	28

* Results are shown as median and percentiles. The distribution of the values was positively skewed (with extreme values/outliers).

Mean serum leptin levels were 20% higher in females (16.39 ng/ml) than males (13.1 ng/ml) in the total sample. The range of leptin levels in females was much wider, with a maximum reaching 204 ng/ml, compared to a maximum of 49 ng/ml in males. Leptin levels correlated weakly with neonatal birth weight (r = 0.14, P<0.05) and length (r = 0.13, P<0.05). The correlations were relatively stronger for males (r = 0.18, P<0.05 for leptin vs weight and r = 0.19, P<0.05 for leptin vs length) than females (r = 0.12, P<0.05 for leptin vs weight, no significant correlation for leptin vs length).

The 90^th^ percentile (28 ng/ml) was considered as the upper limit of normal leptin levels in neonates.

**Figure 2:**
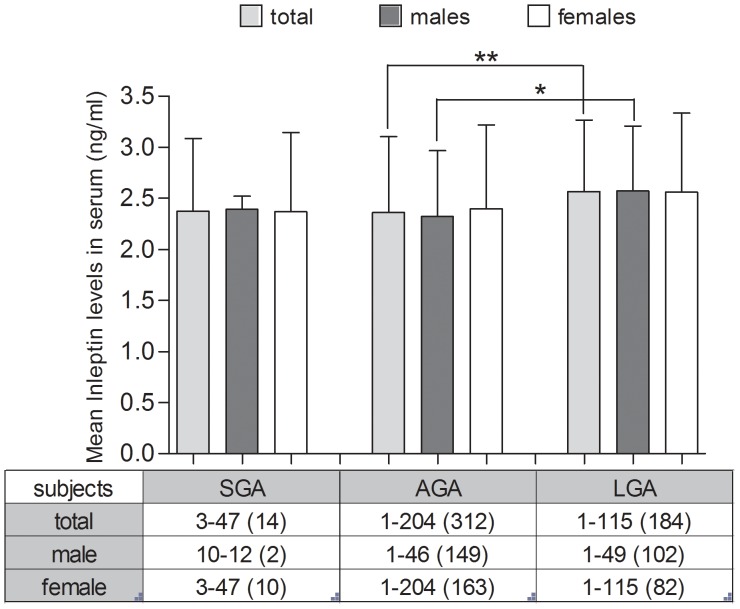
Cord blood (ln)leptin levels in SGA, AGA and LGA neonates. Values show mean ± SD. Statistically significant differences are indicated by asterisks (*, P<0.05; **, P<0.01, compared with the Kruskal-Wallis test). The table shows the min. and max. values (ng/ml) and the number of subjects (n) per group (total, males, females).

Figure 2 shows the leptin (ln) mean differences between SGA, AGA and LGA neonates. Leptin (ln) differences between AGA and LGA neonates were statistically significant (P<0.005). LGA neonates had higher leptin levels (mean 2.57 ng/ml) than AGA neonates (mean 2.36 ng/ml). LGA males had statistically significant higher mean leptin levels than AGA males (P<0.05). There were no statistically significant differences between AGA and SGA neonates or LGA and SGA neonates.

### Analysis of Neonates with High Leptin Levels

**Figure 3:**
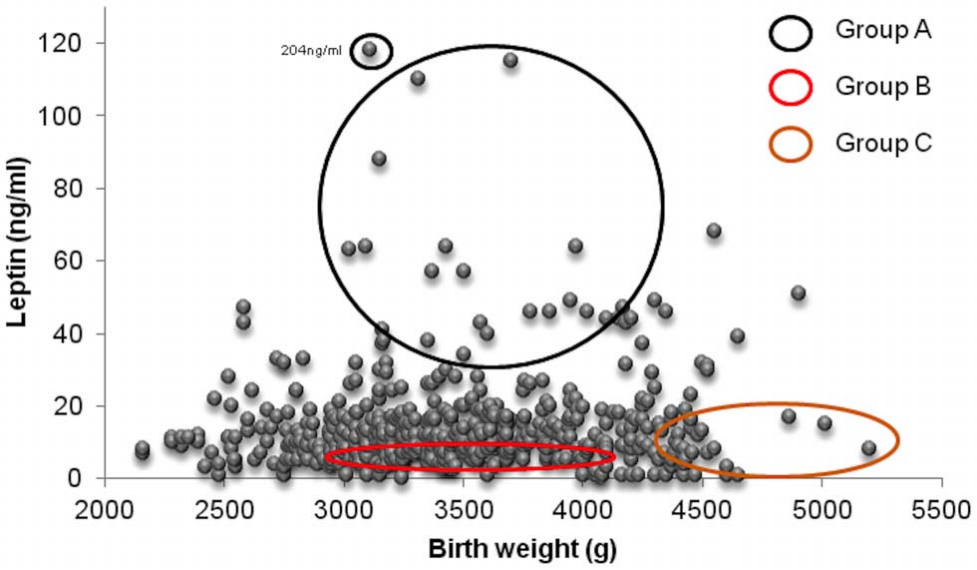
Scatter plot of cord blood leptin levels vs birth weight to show the populations from which the study groups A, B and C were selected and analyzed further for serum cytokine levels. The black circle includes 19 neonates with mean leptin levels over the 90^th^ percentile (group A), the red circle includes 17 neonates with mean leptin levels around the 50^th^ percentile (group B) and the orange circle includes 20 neonates with mean leptin level below the 10^th^ percentile.

In 11.2% neonates (1 SGA, 32 AGA, 24 LGA), leptin levels were well above the 90^th^ percentile, with a median of 39 ng/ml. In a proportion of these neonates (33.3%, 3.72% of the total sample) leptin levels reached max. values (median 46 ng/ml, mean 58.11 ng/ml). To study this group further, we defined 3 groups within our study population (Fig. 3): Group A, consisting of neonates with leptin levels >90^th^ percentile, group B, consisting of neonates with leptin levels around the 50^th^ percentile (median 12 ng/ml, mean 12.41 ng/ml) and group C, consisting of neonates with leptin levels below the 10^th^ percentile (median 1 ng/ml, mean 6.45 ng/ml).

**Figure 4:**
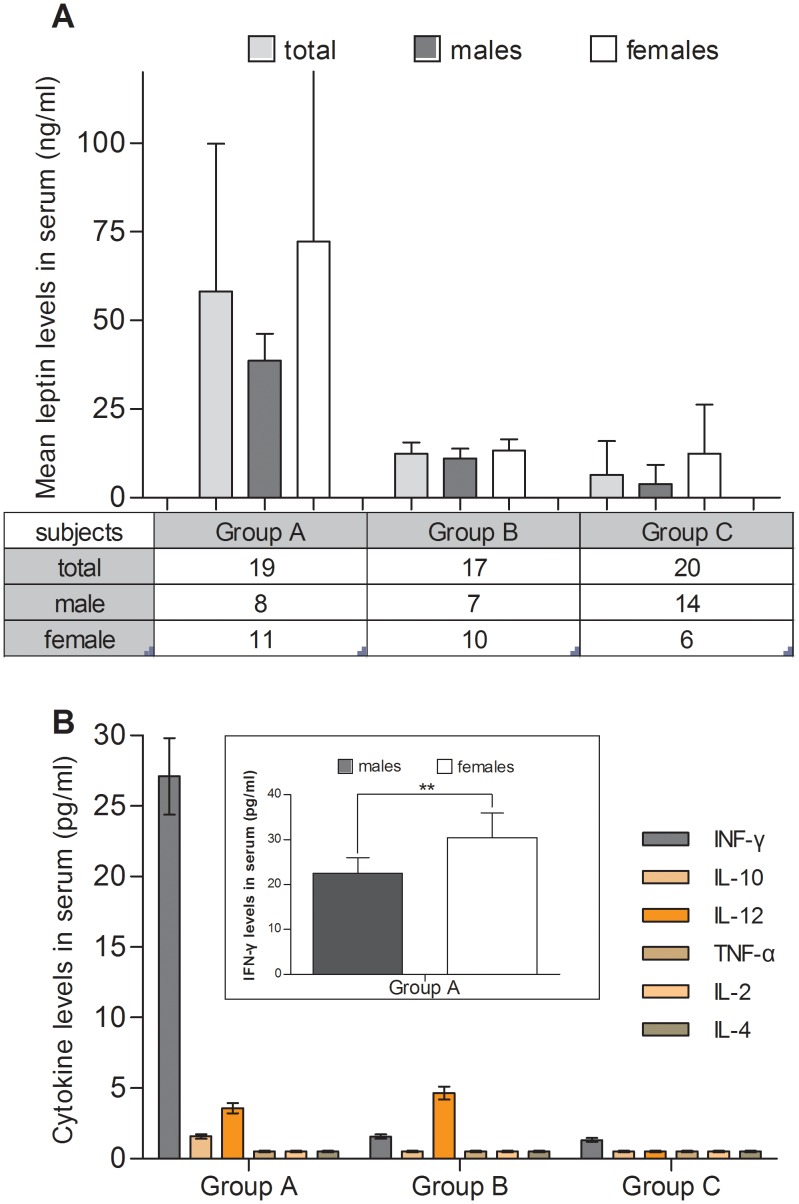
Leptin and cytokine levels in serum. (A) Cord blood leptin levels (ng/ml) in serum of study groups A (n = 19), B (n = 17) and C (n = 20) in the total sample, males and females (mean ± SD). (B) Cytokine serum levels (pg/ml) in groups A, B and C (mean ± SD). (B insert) IFN-γ serum levels in male and female neonates of group A. Statistically significant differences are indicated by asterisks (**, P<0.005, compared with t-test).

Cord blood serum samples were selected from these 3 groups for further analysis. The anthropometric characteristics of the neonates from which these samples were derived are described in Table 5. The leptin levels of groups A, B and C are shown in Figure 4A.

**Table 5:** Anthropometric characteristics of study groups A, B and C*.

	Group A			Group B			Group C		
Variables	Total(n = 19)	Male(n = 8)	Female(n = 11)	Total(n = 17)	Male(n = 7)	Female(n = 10)	Total(n = 20)	Male(n = 14)	Female(n = 6)
Birth wt. (g)	3403	3535	3307	3558	3548	3566	4258	4219	4348
Length (cm)	52.03	53.08	51.27	50.68	51.36	50.2	54.12	53.96	54.5
Head circ. (cm)	34.81	35.68	34.18	34.45	34.71	34.27	36.01	35.94	36.16
PI	2.41	2.35	2.45	2.72	2.61	2.8	2.69	2.7	2.69

* Descriptive statistics performed to describe the anthropometric characteristics of the A, B and C groups of neonates studied. Data variability for the groups are presented as mean.

### Serum Cytokine Levels of Groups A, B and C

The cytokines IL-2, IFN-γ, IL-4, IL-10, IL-12 and TNF-α, were measured in the sera of groups A, B and C (Figure 4B). In group A, significantly elevated levels of serum IFN-γ were found (mean 27.11 pg/ml, range 17.5–38.5 pg/ml). Group A female neonates had higher serum levels of IFN-γ than males (30.5±5.5 pg/ml vs 22.5±3.5 pg/ml, P<0.005) (Figure 4B, insert). IFN-γ in groups B and C was in trace amounts (0–4.44 pg/ml). In all 3 groups, the levels of IL-2, IL-4, IL-10, IL-12 and TNF-α were below or around the detection limits of the assays employed for their detection (M&M).

**Figure 5:**
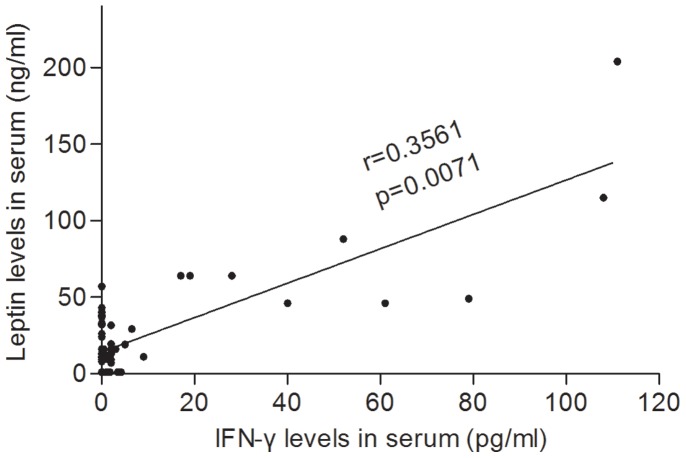
Leptin levels correlated with IFN-γ levels, r = 0.356, P<0.01 (Spearman’s r for non parametric values). There was no correlation between leptin and the other cytokines tested. Correlation is significant at the P<0.01 level (2-tailed).

Leptin levels correlated with IFN-γ levels (r = 0.356, P<0.01 for leptin vs IFN-γ) (Figure 5). There was no correlation between leptin and the other cytokines tested (Supplementary Table S1). To confirm the correlation between leptin and IFN-γ and investigate whether any of the other cytokines interfere with leptin and vice versa, we run a multivariable analysis using the General Linear Model (cf. M&M). The analysis (Supplementary Table S2) confirmed that only the interaction between IFN-γ and leptin was statistically significant (P<0.001).

### Effect of Rleptin on Cytokine Gene Expression in Cord Blood Mononuclear Cells (CBMC) vs Adult PBMC

**Figure 6:**
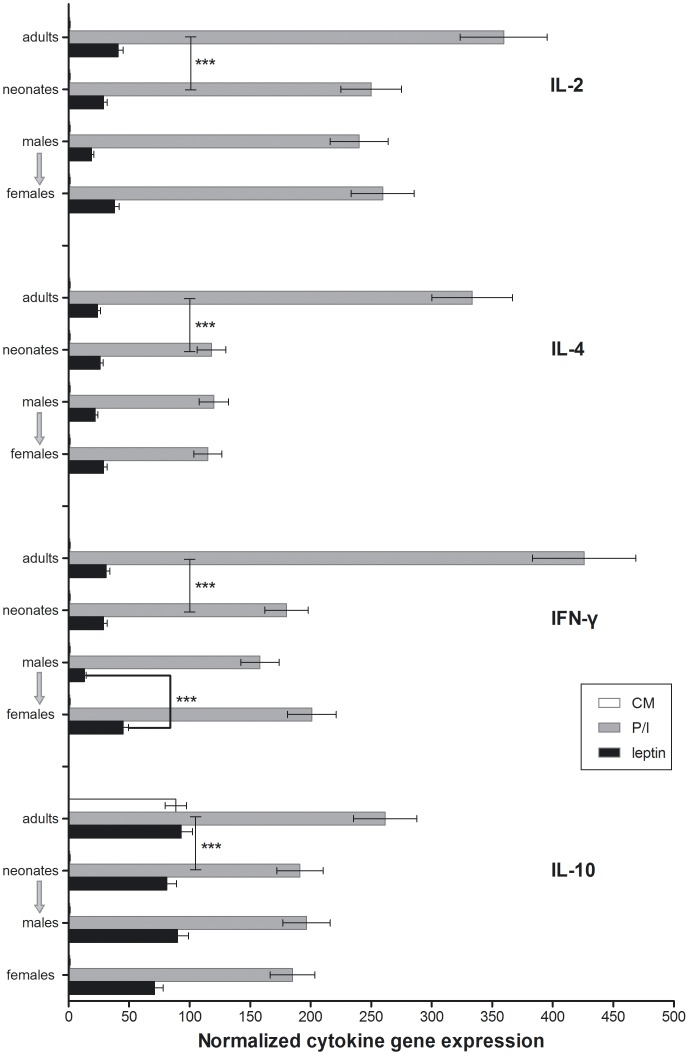
Cytokine gene expression (mean ±%CV) by CBMC of 40 healthy neonates and PBMC of 20 adults. The cells were cultured for 8h in plain culture medium (CM) or with mitogens (P/I) or with recombinant leptin (leptin). The levels of expression were normalized according to β2m expression levels. Statistically significant differences are indicated by asterisks (***, P<0.001, compared with 2-way ANOVA).

Figure 6 depicts the results from RT-PCR performed on CBMC and adult PBMC cultured for 8h in plain culture medium (CM) ± mitogens (P/I) ± rleptin (leptin), run under exactly the same PCR conditions and normalized according to β2 microglobulin expression levels (M&M). After 8h of culture in CM, CBMC expressed none of the cytokine genes tested (IL-2, IFN-γ, IL-4, IL-10) whereas adult PBMC expressed IL-10. Both cb and adult mononuclear cells expressed all the cytokine genes tested when cultured with P/I, but cb cells at significantly lower levels. Culture with rleptin induced the expression of IL-2, IFN-γ and IL-4 genes in both cb and adult cells at comparable levels, and of IL-10 in CBMC. IFN-γ expression was significantly higher in CBMC from female neonates cultured with rleptin.

**Figure 7:**
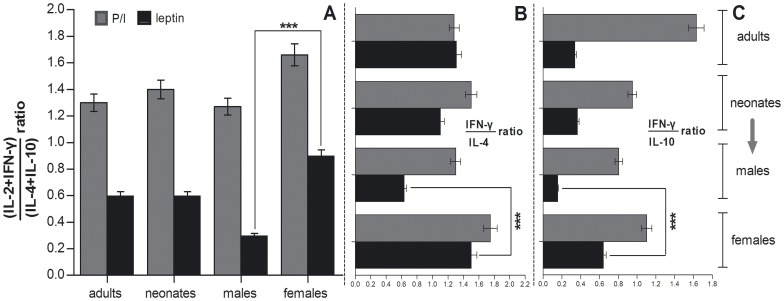
Cytokine gene expression ratios in 40 neonate CBMC (male and female) vs 20 adult PBMC cultured with P/I or rleptin. A, type-1 (IL-2+ IFN-γ) over type-2 (IL-4+ IL-10) gene expression ratios; B, Th1 (IFN-γ) over Th2 (IL-4) gene expression ratios; C, Th1 (IFN-γ) over IL-10 gene expression ratios. Results are given as mean ±%CV; asterisks indicate statistical significance (***, P<0.001, compared with 2-way ANOVA).

Figure 7 shows the type-1 cytokine (IL-2, IFN-γ) over type-2 cytokine (IL-4, IL-10) gene expression ratio (Fig. 7A), Th1 (IFN-γ) over Th2 (IL-4) gene expression ratio (Fig. 7B), and Th1 (IFN-γ) over IL-10 gene expression ratio (Fig. 7C) in adult PBMC and CBMC cultured with P/I or rleptin. Type-1/type-2, Th1/Th2 and Th1/IL-10 ratios were significantly higher in cb female samples cultured with rleptin than in cb male samples, and comparable to adult levels.

### Effect of Rleptin on Cytokine Gene Expression in Purified Cord Blood or Adult Peripheral Blood T-cells and Monocytes

Table 6 depicts the results from RT-PCR performed on purified cb and adult CD3+ T-cells and CD14+ monocytes cultured ± leptin and processed in parallel. Culture with rleptin induced IL-2, IFN-γ and IL-4 gene expression in T-cells and IL-10 gene expression in monocytes, in different sample proportions. Leptin induced the expression of the cytokine genes in a higher number of female than male cb T or Mc cultures. The values obtained for gene intensities were comparable, with the exception of IFN-γ that was significantly higher in female cb T-cells than male cb T-cells.

**Table 6:** Effect of rleptin on cytokine gene expression in cord blood and adult peripheral blood T-cells and monocytes≠.

			Cytokine genes expressed			
Samples		Culture	IL-2	IFN-γ	IL-4	IL-10
cb T-cells (n = 40)	♀ (n = 20)	CM	0	0	0	0
		+ rleptin	8 (29–185)	6 (121–170)**^#^**	6 (80–122)	0
	♂ (n = 20)	CM	0	0	0	0
		+ rleptin	4 (45–136)	4 (40–102)**^#^**	4 (43–182)	0
cb monocytes (n = 40)	♀ (n = 20)	CM	0	0	0	0
		+ rleptin	0	0	0	14 (42–233)
	♂ (n = 20)	CM	0	0	0	0
		+ rleptin	0	0	0	12 (27–366)
adult PB T-cells (n = 20)		CM	0	0	0	0
		+rleptin	4(192–219)	6 (68–161)	4 (100–148)	0
adult PB monocytes (n = 20)		CM	0	0	0	6**^¥^** (172–397)
		+rleptin	0	0	0	+4*(102–284)

≠ Results from RT-PCR performed on purified cb and adult CD3+ T-cells and CD14+ monocytes cultured for 8h ± rleptin. The numbers denote positive samples; in parentheses, the range of gene intensities in pixels for the positive samples (**^#^**Students’ t-test, P<0.05). Culture, culture conditions; cb, cord blood; PB, peripheral blood; n, number of samples; CM, plain culture medium;

* adult monocyte samples that did not express any of the cytokine genes tested in CM and responded to rleptin; rleptin did not affect the IL-10 expression of the 6 positive samples in CM (**^¥^**).

Phenotypic analysis showed that cb CD3+ T-cells consisted of 35.6±5% T helper cells (CD3+CD4+) of which 90.5±7% were naive (CD45RA+), and 42.4±15.1% T cytotoxic cells (CD3+CD8+) of which 89.5±10% were naive. Phenotypic analysis of adult CD3+ T-cells showed that they consisted of 47±6% T helper cells of which 42±7% were naive, and 24±7% T cytotoxic cells of which 75±15% were naive. Gender differences were not observed in the phenotypic sub-analyses (not shown).

Leptin induced IL-10 expression in 60% of male and 70% of female cb-Mc samples. Cb-Mc did not express IL-10 after 8h culture, whereas 30% of adult-derived Mc expressed IL-10 after 8h culture. Leptin did not superinduce IL-10 in the IL-10-positive adult Mc samples, but induced IL-10 in 20% of the IL-10-negative adult Mc samples.

## Discussion

In a relatively large sample of healthy newborns born to healthy mothers, we found no significant correlation between cord blood leptin levels and anthropometric characteristics of the mothers. Male and female neonates had similar somatometric characteristics and PI at birth. Overall, leptin levels correlated with birth weight and length; the correlations were relatively stronger for males than females, in accordance to [41]. Leptin levels were also higher in females than males; nevertheless, the difference between mean leptin levels in male neonates (13.1±9.37 ng/ml) and female neonates (16.39±19.6 ng/ml) in the whole sample did not reach statistical significance, due to the large SD values in the females. This was reflected in all groups for female neonates i.e. SGA, AGA and LGA. The number of SGA infants included in this study was much smaller than AGA and LGA infants, because we set strict inclusion criteria for neonates, free of any risk factors (M&M), and for these reasons our findings in leptin levels between SGA and AGA or LGA populations did not reach statistical significance. Significantly lower leptin levels have been recorded for SGA infants, compared to AGA, in a study cohort of 190 infants (29 SGA, 161 AGA), that included 52% pre-term and low birth weight infants, of which 22% were SGA [42].

Interestingly, although leptin levels were higher in LGA vs AGA neonates, in accordance to [43], only 9.6% of AGA neonates had leptin levels below the 10^th^ percentile compared to 16.3% of LGA neonates. Overall, a higher proportion of LGA neonates had lower leptin levels than AGA neonates. If leptin levels in cord blood reflect the rate of leptin synthesis by fetoplacental tissues, then leptin may be a factor that regulates growth of the fetus and nutrient partitioning so, in this sense, higher adiposity in LGA healthy infants may result from lower leptin production [44].

In a small proportion of neonates (3.72% of the total sample), with the highest leptin levels (group A, median serum leptin concentration 46 ng/ml), IFN-γ serum levels at birth were elevated (range 17.5–38.5 pg/ml) whereas the rest of the cytokines tested were very low (below or around the detection limits of the assays, cf. M&M). In addition, group A female neonates had significantly higher serum levels of IFN-γ than males. We did not observe increased levels of any of the cytokines tested in the sera of neonates with average (group B) or very low (group C) leptin levels. Elevated serum levels of pro-inflammatory cytokines and, in particular IFN-γ have not been reported for healthy term or near-term infants; rather, elevated cord blood serum levels of IFN-γ and other pro-inflammatory cytokines have been observed in premature infants that developed respiratory distress syndrome soon after birth, as we have shown recently [45].

The medical records of group A newborns and maternal notes were reviewed for any additional information about their intrauterine and perinatal period. It was confirmed that these newborns were free from any environmental or genetic risk factor and no intrauterine or perinatal infection were recorded; 15 were delivered normally and 4 by elective caesarean section.

Experiments carried out to delineate the direct effect of leptin on cord blood-derived mononuclear cells, purified T-cells and monocytes, in comparison with the same cell types derived from healthy adults, showed that activation of the cells by P/I resulted in significantly lower expression of the cytokine genes in CBMC compared to adult PBMC. In addition, cb monocytes did not express any of the cytokine genes tested when cultured for 8h in plain CM, whereas PBMC-derived monocytes expressed IL-10. These results support the conclusions of published reports showing that neonatal immunity is relatively impaired compared to adult immunity [46], [47], [48], [49], [50]. In contrast, CBMC were activated by leptin to express IL-2, IFN-γ, IL-4 and IL-10 genes at comparable levels to adult PBMC.

When CBMC were analysed for cytokine gene expression separately for female and male neonates, a difference was observed in the expression levels of IFN-γ that was significantly higher in female CBMC and in purified female cb T-cells. This was an intriguing finding in the light of recent research demonstrating that IFN-γ acts directly on adipocytes inducing them to release inflammatory chemokines and cytokines [51], [52].

Experiments with purified T-cells and monocytes showed that leptin induced the expression of IL-2, IFN-γ and IL-4 genes in T-cells and IL-10 in monocytes in both CBMC and PBMC samples. Nevertheless, quantitative differences were observed, i.e. cb monocytes responded better to leptin in culture than PBMC monocytes, and a higher proportion of female cb T-cells responded to leptin than male cb or PBMC T-cells. These data cannot be explained by the naive/memory T-cell dichotomy proposed earlier [31], because the phenotypic analysis performed to describe the cb and adult T-cells analyzed in this study, showed that all adult T-cell samples contained a substantial proportion of naive T helper and cytotoxic cells, and, also, gender differences were not observed in the phenotypic sub-analyses. We therefore suggest that the effect of leptin on T-cells is more complicated than previously thought, and future experiments with highly purified T-cell populations isolated from cord blood or adult PBMC will enable us to understand better the biochemical and molecular pathways involved in leptin’s effect on neonatal and adult adaptive immunity.

**Figure 8:**
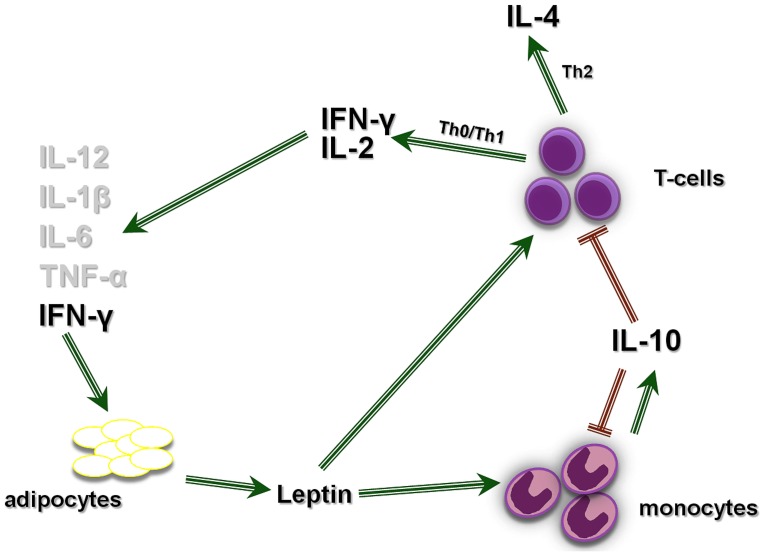
Proposed model of leptin regulation of cytokine production by cord blood T-cells and monocytes. Leptin is produced by adipocytes (and other tissues including the placenta), binds to its receptor (OB-R) on T-cells and monocytes [29], and directly activates them to express cytokines. T-cells express the Th0/Th1 cytokine genes IFN-γ and IL-2 and the Th2 cytokine gene IL-4, that mediate effector functions [57], and monocytes express IL-10, a main anti-inflammatory cytokine that limits the immune response and prevents damage to the host [58]. In infants with very high leptin levels, leptin stimulation of IFN-γ production by T-cells overrides IL-10 production by monocytes [59]; in this milieu, a Th1 response predominates; in addition, IFN-γ may enhance cytokine stimulation of leptin synthesis by adipocytes [51], [52], [60], [61], [62] and perpetuate the Th1 polarization.

In Figure 8 we propose a model that shows the cross-regulation of leptin and cytokine production in cord blood. The significantly increased IFN-γ expression of female CBMC or T-cells when cultured with rleptin, indicate that high leptin levels in the neonate may create a Th1 polarization that, in females, may contribute to a propensity to develop T-cell-mediated autoimmunity later in life, as others have shown for hormones [53] or the expression levels of the nuclear receptor peroxisome-proliferator-activated receptor-α in T-cells [54]. To note, the max. values of serum leptin in group A neonates were measured in females, with a maximum reaching 204 ng/ml, whereas the max. value for males was 49 ng/ml. On the other hand, the production of IFN-γ by cord blood cells may protect infants from allergic conditions [46] or susceptibility to the infections of childhood [47].

On follow up, interviews were carried out by phone with the parents, or those who had parental responsibility, to enquire about the health status of group A children. It was reported that all group A children were healthy and fully immunized. Their weight and height were between the 50^th^ – 75^th^ percentiles on the growth charts. Detailed follow up of the children, originally entered in the study as neonates, may reveal more aspects of leptin’s role.

## Materials and Methods

### Study Subjects

Five hundred and ten (510) healthy infants of singleton pregnancies were studied. Selection criteria included uncomplicated perinatal period and term or near term birth (gestational age over 36 weeks and with a birth weight >2,150 g). Three hundred and twelve (312) were AGA infants and 184 were LGA. Fourteen (14) SGA infants with no further abnormalities were also included. These SGA neonates were symmetrical, born at term or near term by normal delivery and they did not need admission to special care unit. They were bottle fed and stayed on the postnatal ward to establish feeds for 3 days.

From the medical records 10 of them were born to multigravida mothers and 4 to primigravida. Maternal booking bloods were negative for infections. Membranes ruptured <18 hours and mother was free of any infection (afebrile, GBS negative, uncomplicated postnatal period). Placenta appeared anatomically normal. Babies never needed resuscitation at birth or screening for sepsis. Babies were discharged home under the care of the community midwife. No subsequent readmissions were recorded in the following 28 days. Other SGA infants were not included in the study because they were regarded as high risk neonates.

Infants with anomalies or who required intensive care were also excluded. Infants whose mothers suffered from any medical complication (including diabetes), who were on any medication (with the exception of iron supplements and vitamins), abused alcohol, drugs or smoked during their pregnancy were excluded.

Birth weight was recorded at birth, using a standard electrical scale by the attending midwife. Height and head circumference were determined within the first 24h of life by a single examiner. Ponderal index (PI) was calculated as an additional postnatal variable for intrauterine growth. PI was calculated as the ratio of the birth weight in grams times 100 to cubed value of the length in cm. The intrauterine status of the infants was categorized as SGA (birth weight below the 10^th^ percentile), AGA (birth weight between 10^th^ and 90^th^ percentiles) and LGA (birth weight higher than the 90^th^ percentile) [55]. Newborn gestational age was determined according to Ballard’s scoring system, assuming two weeks as a reasonable error of the estimated gestational age based upon the first day of the last menstrual period and supported by ultrasound measurements.

Twenty healthy adult volunteers served as controls. They were 12 F, age 27–34 years, BMI 17.3–21.7, serum leptin (ng/ml): mean ± SD 8.8±5.0, median 7.4, and 8M, age 25–56 years, BMI 23.6–26.1, serum leptin (ng/ml): mean ± SD 6.4±1.9, median 7.3.

Adult controls and the women before labor provided written informed consent for the use of their peripheral blood samples or their childrens’ cord blood samples for the purpose of this research, or, if they were unable to read or write, independently witnessed verbal consent was obtained. The study was submitted to and approved by the internal review board and scientific advisory committee of Patras University Hospital, as part of a general application for studies on «the role of cytokines and hormones in the pathogenesis of autoimmune diseases», entailing the use of the study subjects’ clinical data to be used (unamed) in the sections of «Patients, Materials & Methods» and of the blood samples to be used in *in vitro* experiments to be described in the resulting publications. The Hospital abides by the Helsinki declaration on ethical principles for medical research involving human subjects.

### Determination of Serum Leptin and Cytokine Concentrations

The umbilical cord was clamped immediately and umbilical cord blood samples were obtained from the vein, just after an uncomplicated delivery (normal or by elective cesarean section). The serum was separated, aliquoted and stored at −80°C. Serum leptin and cytokine IL-12 levels were measured by ELISA (R&D systems, Minneapolis, USA). The detection limits of the assays were 7.8 pg/ml for leptin and 5 pg/ml for IL-12. Cytokine IFN-γ, TNF-α, IL-2, IL-4 and IL-10 levels were measured by a cytometric bead array (CBA) assay (BD Biosciences, San Diego, USA) for the detection of individual cytokines in sera (flex sets) and analyzed on a BD FACSArray Bioanalyzer. The detection limits of the assays were 1.8 pg/ml for IFN-γ, 1.2 pg/ml for TNF-α, 11.2 pg/ml for IL-2, 1.4 pg/ml for IL-4 and 0.13 pg/ml for IL-10. All samples were analyzed in duplicate.

### Cell Isolation, Phenotyping and RT-PCR

Blood samples, cord blood (15–25 ml) from 40 neonates and peripheral blood (10 ml) from 20 adults, were collected in heparinized tubes. Mononuclear cells were isolated by centrifugation over a Ficoll-Paque gradient (Biochrom AG, Berlin, Germany). For isolation of T-cells and monocytes, mononuclear cells were washed 4x with ice-cold RPMI1640 culture medium (Gibco BRL, Gaithersburg, MD) and CD3+ T-cells or CD14+ monocytes were isolated using antibody-coated magnetic bead kits for the isolation of untouched human T-cells or monocytes from PBMC, at a concentration of 10^6^ cells/ml, 2 beads/cell (Dynabeads®, Invitrogen, Biotech ASA, Oslo, Norway). The CD3+ T-cells and CD14+ monocytes were tested by FACS for purity (all samples were >95% pure) with the mouse anti-human monoclonal antibodies: CD3-PE-Cy5 (Beckman Coulter, France) and CD14-FITC (BD Biosciences/Pharmingen, San Diego, CA, USA). CD3+ T-cells were phenotyped further to estimate their content of naive (CD45RA+) T-cells, in the T helper (CD4+) and T cytotoxic (CD8+) cell subsets. The antibodies used were mouse anti-human monoclonal CD4-FITC (BC), CD8-FITC (BC), CD45RA-PE (BD). CD3+ T-cells and CD14+ monocytes were cultured at a concentration of 10^6^ cells/ml for 8h either in plain culture medium (CM) or with the mitogens phorbol myristate acetate (20 ng/ml) and ionomycine (2 µΜ) (P/I) or with recombinant (r) leptin (800 ng/ml) [28]. At the end of the culture period, total RNA was isolated from the cells. One (1) µg of total RNA/sample was reverse transcribed to cDNA using random primers, and RT-PCR was performed at 35 cycles to determine cytokine gene expression levels with specific primers for IL-2, IL-4, IFN-γ and IL-10, using β2 microglobulin mRNA as an endogenous control [56]. The results were quantified by measuring the intensity of the PCR products after agarose gel analysis by the use of the Scion Image program. The levels of expression were normalized according to β2m expression levels. All measurements were done in triplicate.

### Statistical Analysis

Serum leptin values were not normally distributed and the data are presented as median (range). The data were normalized by log-transformation and Spearman’s correlations were used to evaluate any relationship among the variables of interest. The rest of the data were normally distributed and are presented as mean ± SD. The differences in baseline characteristics (gestational age, maternal age, ethnicity and gender) were examined by two-way analysis of variance. The differences in newborn’s anthropometric measures and cord blood serum leptin concentration (after ln transformation as appropriate) were examined using a two-way analysis of covariance. Multiple linear regression analysis was used to examine relationships between somatometric and demographic characteristics of the mothers and cord blood leptin levels of the newborns, and between cord blood leptin and cytokine levels. All corrections for multiple comparisons were done with the Bonferroni procedure. Confirmation of correlations was done using the General Linear Model. The Statistical Package for Social Sciences (SPSS), version 17, was used for statistical analysis.

## Supporting Information

Table S1 Matrix representing the correlation between all variables (cytokines and leptin)^#^. ^#^Spearman’s r for non parametric values. **Correlation is significant at the P<0.01 level (2-tailed).
(DOCX)

Table S2 Tests of Between-Subjects Effects^#^. ^#^Corrected model showing that only the interaction between the IFN-γ and leptin is statistically significant (p<0.001). a, R Squared = 0.242 (Adjusted R Squared = 0.214); b, R Squared = 0.000 (Adjusted R Squared = 0.000); c, R Squared = 0.126 (Adjusted R Squared = 0.093); d, R Squared = 0.032 (Adjusted R Squared = −0.004); e, R Squared = 0.469 (Adjusted R Squared = 0.499).
(DOC)
