# Supplementary material for: Cord Blood Leptin Levels of Healthy Neonates Are Associated with IFN-γ Production by Cord Blood T-Cells
Source: PLoS One. 2012 Jul 16;7(7):e40830. doi: 10.1371/journal.pone.0040830 (PMC3397963; doi:10.1371/journal.pone.0040830)
Supplement: Table S1 — Matrix representing the correlation between all variables (cytokines and leptin)#. #Spearman’s r for non parametric values. **Correlation is significant at the P<0.01 level (2-tailed). (DOCX) [file pone.0040830.s001.docx]

**Table S1.** Matrix representing the correlation between all variables (cytokines and leptin)^#^

|  |  | leptin | IFN-γ | IL-2 | TNF- α | IL-10 | IL-4 | IL-12 |
| --- | --- | --- | --- | --- | --- | --- | --- | --- |
| **leptin** | Correlation coefficient | 1.000 | **0.356**** | - | - | -0.127 | - | 0.194 |
|  | Sig.(2-tailed) | - | **0.007** | - | - | 0.351 | - | 0.151 |
|  | N | 56 | 56 | 56 | 56 | 56 | 56 | 56 |
| **IFN-γ** | Correlation coefficient | **0.356**** | 1.000 | - | - | 0.109 | - | 0.050 |
|  | Sig.(2-tailed) | **0.007** | - | - | - | 0.426 | - | 0.717 |
|  | N | 56 | 56 | 56 | 56 | 56 | 56 | 56 |
| IL-2 | Correlation coefficient | - | - | - | - | - | - | - |
|  | Sig.(2-tailed) | - | - | - | - | - | - | - |
|  | N | 56 | 56 | 56 | 56 | 56 | 56 | 56 |
| TNF-α | Correlation coefficient | - | - | - | - | - | - | - |
|  | Sig.(2-tailed) | - | - | - | - | - | - | - |
|  | N | 56 | 56 | 56 | 56 | 56 | 56 | 56 |
| IL-10 | Correlation coefficient | -0.127 | 0.109 | - | - | 1.000 | - | -0.177 |
|  | Sig.(2-tailed) | 0.351 | 0.426 | - | - | - | - | 0.192 |
|  | N | 56 | 56 | 56 | 56 | 56 | 56 | 56 |
| IL-4 | Correlation coefficient | - | - | - | - | - | - | - |
|  | Sig.(2-tailed) | - | - | - | - | - | - | - |
|  | N | 56 | 56 | 56 | 56 | 56 | 56 | 56 |
| IL-12 | Correlation coefficient | 0.194 | 0.050 | - | - | -0.177 | - | 1.000 |
|  | Sig.(2-tailed) | 0.151 | 0.717 | - | - | 0.192 | - | - |
|  | N | 56 | 56 | 56 | 56 | 56 | 56 | 56 |
