# Supplementary material for: Cord Blood Leptin Levels of Healthy Neonates Are Associated with IFN-γ Production by Cord Blood T-Cells
Source: PLoS One. 2012 Jul 16;7(7):e40830. doi: 10.1371/journal.pone.0040830 (PMC3397963; doi:10.1371/journal.pone.0040830)
Supplement: Table S2 — Tests of Between-Subjects Effects#. #Corrected model showing that only the interaction between the IFN-γ and leptin is statistically significant (p<0.001). a, R Squared = 0.242 (Adjusted R Squared = 0.214); b, R Squared = 0.000 (Adjusted R Squared = 0.000); c, R Squared = 0.126 (Adjusted R Squared = 0.093); d, R Squared = 0.032 (Adjusted R Squared = −0.004); e, R Squared = 0.469 (Adjusted R Squared = 0.499). (DOC) [file pone.0040830.s002.doc]

**Table S2.** Tests of Between-Subjects Effects^#^

| Sourse | Dependent Variable | Type III Sum of Squares | df | Mean Square | F | Significance |
| --- | --- | --- | --- | --- | --- | --- |
| **Corrected Model** | **IFN-γ** | **8275.371^a^** | 2 | **4137.685** | **8.476** | **0.001** |
|  | TNF-α | 0.000^b^ | 2 | 0.000 | - | - |
|  | IL-2 | 0.000^b^ | 2 | 0.000 | - | - |
|  | IL-4 | 0.000^b^ | 2 | 0.000 | - | - |
|  | IL-10 | 222.623^c^ | 2 | 111.312 | 3.822 | 0.028 |
|  | IL-12 | 224.611^d^ | 2 | 112.306 | 0.880 | 0.421 |
| Intercept | **leptin** | **29851.604^e^** | 2 | **14925.802** | **23.441** | **0.000** |
|  | IFN-γ | 5569.321 | 1 | 5569.321 |  |  |
|  | TNF-α | 0.000 | 1 | 0.000 |  |  |
|  | IL-2 | 0.000 | 1 | 0.000 |  |  |
|  | IL-4 | 0.000 | 1 | 0.000 |  |  |
|  | IL-10 | 155.924 | 1 | 155.924 |  |  |
|  | IL-12 | 419.108 | 1 | 419.108 |  |  |
|  | leptin | 36288.128 | 1 | 36288.128 |  |  |
| Controls | IFN-γ | 8275.371 | 2 | 4137.685 |  |  |
|  | TNF-α | 0.000 | 2 | 0.000 |  |  |
|  | IL-2 | 0.000 | 2 | 0.000 |  |  |
|  | IL-4 | 0.000 | 2 | 0.000 |  |  |
|  | IL-10 | 222.623 | 2 | 111.312 |  |  |
|  | IL-12 | 224.611 | 2 | 112.306 |  |  |
|  | leptin | 29851.604 | 2 | 14925.802 |  |  |
| Error | IFN-γ | 25871.343 | 53 | 488.139 |  |  |
|  | TNF-α | 0.000 | 53 | 0.000 |  |  |
|  | IL-2 | 0.000 | 53 | 0.000 |  |  |
|  | IL-4 | 0.000 | 53 | 0.000 |  |  |
|  | IL-10 | 1543.372 | 53 | 29.120 |  |  |
|  | IL-12 | 6766.514 | 53 | 127.670 |  |  |
|  | leptin | 33746.864 | 53 | 636.733 |  |  |
| Total | IFN-γ | 39906.843 | 56 |  |  |  |
|  | TNF-α | 0.000 | 56 |  |  |  |
|  | IL-2 | 0.000 | 56 |  |  |  |
|  | IL-4 | 0.000 | 56 |  |  |  |
|  | IL-10 | 1943.746 | 56 |  |  |  |
|  | IL-12 | 7377.000 | 56 |  |  |  |
|  | leptin | 100419.047 | 56 |  |  |  |
| Corrected total | IFN-γ | 34146.714 | 55 |  |  |  |
|  | TNF-α | 0.000 | 55 |  |  |  |
|  | IL-2 | 0.000 | 55 |  |  |  |
|  | IL-4 | 0.000 | 55 |  |  |  |
|  | IL-10 | 1765.995 | 55 |  |  |  |
|  | IL-12 | 6991.125 | 55 |  |  |  |
|  | leptin | 63598.468 | 55 |  |  |  |
